# An Insight Into Ameliorating Production, Catalytic Efficiency, Thermostability and Starch Saccharification of Acid-Stable α-Amylases From Acidophiles

**DOI:** 10.3389/fbioe.2018.00125

**Published:** 2018-09-28

**Authors:** Deepak Parashar, Tulasi Satyanarayana

**Affiliations:** ^1^Functional Genomic Unit, CSIR-Institute of Genomics and Integrative Biology, New Delhi, India; ^2^Division of Biological Sciences and Engineering, Netaji Subhas Institute of Technology, New Delhi, India

**Keywords:** acidophiles, *Bacillus acidicola*, acid-stable α-amylase, thermostability, starch saccharification

## Abstract

Most of the extracellular enzymes of acidophilic bacteria and archaea are stable at acidic pH with a relatively high thermostability. There is, however, a dearth of information on their acid stability. Although several theories have been postulated, the adaptation of acidophilic proteins to low pH has not been explained convincingly. This review highlights recent developments in understanding the structure and biochemical characteristics, and production of acid-stable and calcium-independent α-amylases by acidophilic bacteria with special reference to that of *Bacillus acidicola*.

## Introduction

Enzyme characteristics such as thermostability, selectivity, solvent tolerance and substrate affinity can be improved through genetic engineering based on the availability of large data on improving these characteristics (Verma and Satyanarayana, 2012; Widersten, 2014; Joshi and Satyanarayana, 2015). Alteration in pH stability is tedious and lacks rational approaches. However, a few reports on enhancing acid stability of α-amylases through protein engineering are available (Liu et al., 2008a, 2012; Yang et al., 2013).

The majority of the enzymes used at commercial scale lack adequate acid stability, thus limiting their applications. For using such enzymes, adjusting pH to their optima is required, which makes the process tedious, expensive and time consuming. In order to overcome the problems, many industries use acid-stable enzymes from fungal sources. Since these lack adequate thermostability, the enzymes get denatured when processes are carried out at elevated process temperatures (Demirjian et al., 2001; Elleuche et al., 2014). In order to overcome these problems, microbes that are capable of tolerating harsh conditions could be exploited for naturally tailored enzymes that are superior to their neutrophililic counterparts for utility under harsh bioprocess conditions (Hough and Danson, 1999; Eichler, 2001; Sharma et al., 2012; Raddadi et al., 2015). It has generally been observed that the enzymes from acidophilic microbes function under their optimal growth conditions (Ferrer et al., 2007), thus find several commercial applications. Furthermore, the study of these enzymes might also enable us to understand the underlying mechanisms to make them functional in extreme acidic conditions (Demirjian et al., 2001). Although several acidophilic microbes have been reported (Table 1), a very few acid-stable amylases have been studied in adequate detail (Matzke et al., 1997; Sharma et al., 2012). In this review, we have explained possible strategies for improving acid-stable α-amylase production to make the process cost-effective.

**Table 1:** Archaeal and bacterial acidophiles.

**Microorganisms**	**Optimum pH**	**Optimum temp**	**References**
**MESOPHILIC BACTERIA**			
*Bacillus acidicola*	4	37	Sharma and Satyanarayana, 2010
*Leptospirillum ferroxidans*	40	1.6	Zhang et al., 2010
*Acidithiobacillus ferrivorans*	29	2.1	Hedrich and Johnson, 2013
*Acidiphilium organovorum*	37	3	Lobos et al., 1986
*Acidiphilium symbioticum*	37	3–4	Bhattacharyya et al., 1990
*Acidiphilium cryptum*	35–40	3	Harrison, 1981
**MODERATELY THERMOPHILIC BACTERIA**			
*Alicyclobacillus acidocaldarius*	60–65	3–4	Mavromatis et al., 2010
*Acidomicrobium ferroxidans*	48	2	Clark and Norris, 1996
*Sulfobacillus acidophilus*	45–48	2	Norris et al., 1996
*Sulfobacillus thermosulfidooxidans*	45–48	2	Golovacheva and Karavaiko, 1978
*Alicyclobacillus acidiphilus*	50	3	Matsubara et al., 2002
*Leptospirilum thermoferrooxidans*	45–50	1.6–1.9	Golovacheva et al., 1992
*Hydrogenobacter acidophilus*	65	3–4	Shima and Suzuki, 1993
*Alicyclobacillus acidoterrestris*	35–55	2–5	Orr et al., 2000
***Archaea***			
*Thermogymnomonas acidicola*	60	3	Itoh et al., 2007
*Sulfurococcus yellowstonii*	60–65	2–2.5	Karavaiko et al., 1994
*Sulfolobus metallicus*	65–68	1–4.5	Huber and Stetter, 1991
*Picrophilus torridus*	60	0.7	Schleper et al., 1995
*Thermoplasma acidophilum*	59	1–2	Darland et al., 1970
*Thermoplasma volcanium*	60	2	Segerer et al., 1988
*Picrophilus oshimae*	60	0.7	Schleper et al., 1995
**HYPERTHERMOPHILIC ARCHAEA**			
*Acidianus infernus*	90	2	Segerer et al., 1986
*Desulfurolobus ambivalens*	81	2.5	Fuchs et al., 1996
*Metalloshaera sedula*	75	1.7	Huber et al., 1989
*Metallsphaera prunae*	75	1–4	Fuchs et al., 1996
*Sulfolobus acidocaldarius*	80	2–3.5	Brock et al., 1972
*Sulfolobus shibatae*	80	3	Grogan et al., 1990
*Sulfolobus yangmingensis*	80	4	Ren-Long et al., 1999
*Sulfolobus tengchongensis*	85	3.5	Xiang et al., 2003
*Sulfurisphaera ohwakuensis*	84	2	Kurosawa et al., 1998
*Acidilobus aceticus*	85	3.8	Prokofeva et al., 2000

## Need for acid-stable amylases

Starch is a ubiquitous reserve polysaccharide in plants and one of the most abundant energy sources. The starch hydrolyzing enzymes such as α-amylases, glucoamylases, α-glucosidases, and pullulanases have become increasingly attractive for starch industries because of increasing demand for sugar syrups (Sharma and Satyanarayana, 2013a; Elleuche et al., 2014). Moreover, starch hydrolyzing enzymes have attracted attention in ethanol production and account for 25 % of the global enzyme market today (Sharma et al., 2016).

**Figure 1:**
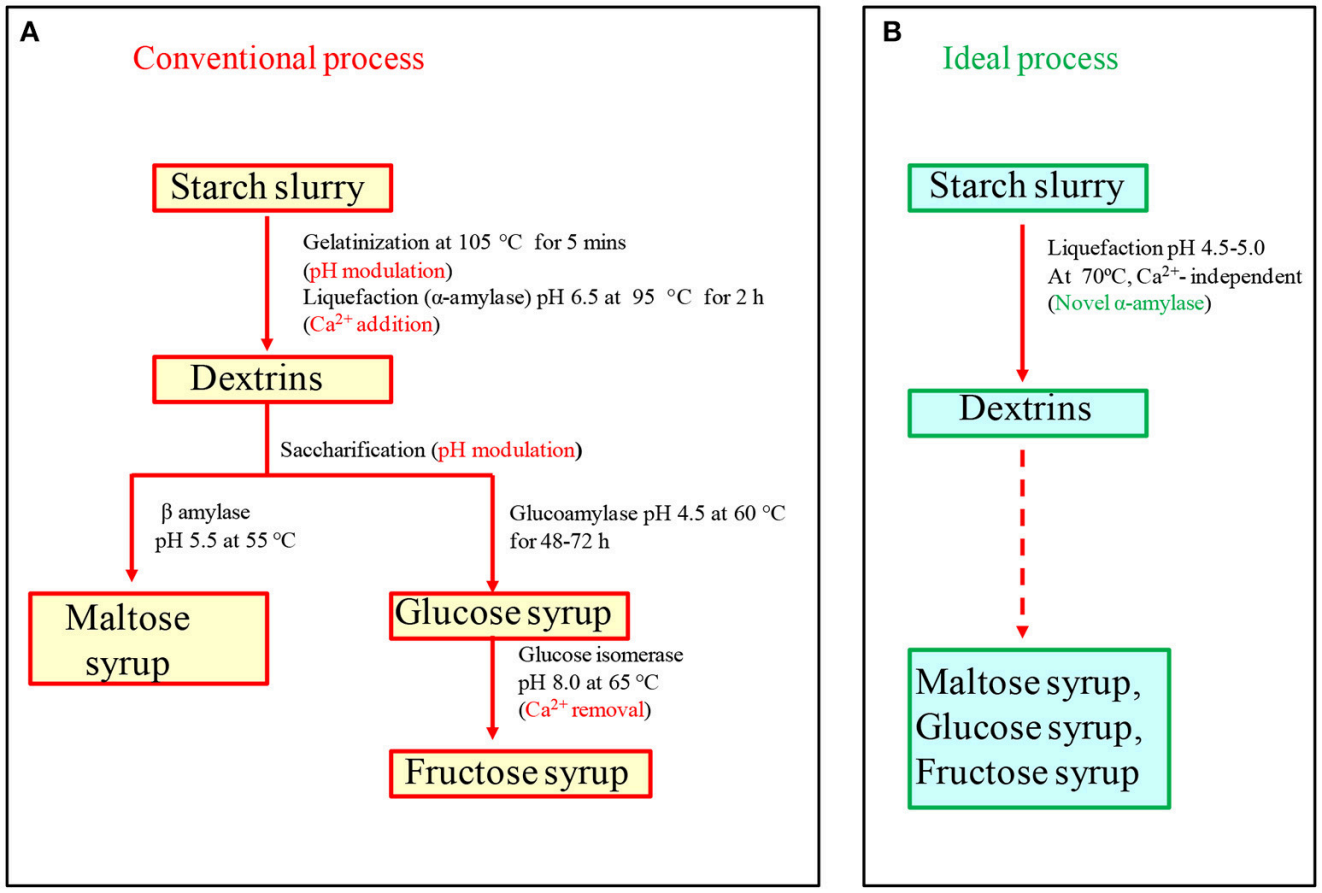
**(A)** Conventional starch saccharification process needs various pH modulation steps and salt addition (Ca^2+^-50 ppm) and removal steps, which increase cost of the process. **(B)** In ideal starch saccharification, pH modulation and calcium addition can be eliminated with the help of acid-stable and Ca^2+^ independent amylase (modified with permission from Sharma and Satyanarayana, 2013a).

The conventional industrial conversion of starch to glucose consists of a three-step industrial process: in the first step, 25–30% starch slurry is gelatinized in a jet cooker at 100–105°C for 5–10 min, in the second step α-amylase and Ca^2+^ (50 ppm) are added with pH adjusted to 6.5 for liquefaction, and in the last step, glucoamylase addition leads to the formation of glucose (Figure 1) (Crabb and Mitchinson, 1997; Mehta and Satyanarayana, 2013). A few bottlenecks are associated with the process: firstly, gelatinization at higher temperature (100–105°C) requires high energy input. Secondly, native pH of starch slurry is around 3–4.5, thus, a pH adjustment step is required because α-amylases that are commercially available function best at pH 6–6.5. Thirdly, most of the α-amylases are Ca^2+^-dependent, and thus, Ca^2+^ is added during the process, which must be removed in the subsequent stages because glucose isomerase used in fructose syrup production is inhibited in the presence of Ca^2+^. In order to make the process economical and time saving, there has been an emphasis on discovering Ca^2+^ independent, acid-stable and raw starch degrading thermostable α-amylases, which can hydrolyze raw starch at sub-gelatinization temperatures bypassing the energy intensive gelatinization, and avoiding Ca^2+^ addition and pH adjustment steps (Sharma and Satyanarayana, 2010; Mehta and Satyanarayana, 2016; Sharma et al., 2016).

## Acidophiles and their biology

Among extreme environments, acidic environments are especially interesting because the low pH of the habitat is a consequence of microbial metabolic activities (e.g., production of acid, deamination of amino acids etc.), and not a condition imposed by the system as in other extreme environments like temperature, radiation and pressure. For example, the extreme conditions of the Tinto River in Southwestern spain are due to the products of metabolic activity of chemolithotrophic microorganisms residing in its water, but not due to the intensive mining activity carried out in the area as believed earlier (Johnson, 1998; González-Toril et al., 2003). Acidophiles are classified as organisms which can withstand and even thrive in acidic environments having pH values in the range of 1.0 to 5.0. Acidophiles are found in eukaryotes (fungi) as well as prokaryotes (bacteria and archaea) which thrive in a variety of acidic environments, including sulphuric pools and geysers, areas polluted by acid mine drainage, and even our own guts (Baker-Austin and Dopson, 2007; Sharma et al., 2012). Based on the optimum temperature required for growth, acidophiles have been subdivided further into various groups: mesophilic acidophiles (*Acidithiobacillus, Ferroplasma, Leptospirillum*), moderate thermoacidophiles (*Picrophilus torridus*) and hyper thermoacidophiles (*Acidianus infernus*) (Table 1). It has, however, been observed that the most thermotolerant microbes are not the most acid tolerant and vice versa (Auernik et al., 2008). The most thermophilic extreme thermoacidophilic archaeon, *Acidianus infernus* that grows at 65–95°C (T_opt_ 90°C), grows in the pH range between 5.5 and 1.0, with the optimum around 2.0 (Segerer et al., 1986). The members of archaeal Picrophilaceae are the most acidophilic organisms known and are able to grow at pH 0.7 and 60°C (Schleper et al., 1995).

**Figure 2:**
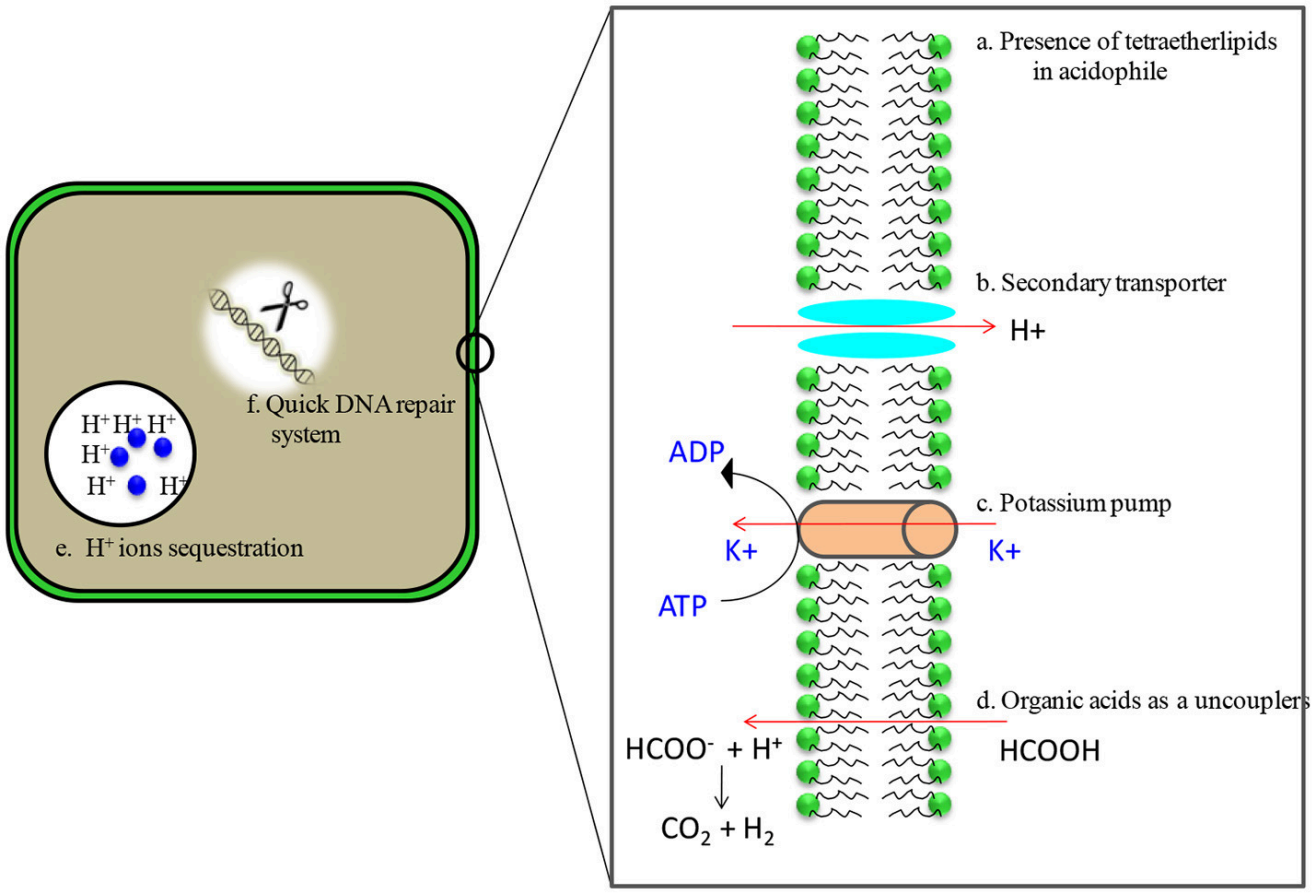
Adaptations of acidophilic microbes in acidic environments. **(a)** membranes are characterized by the presence of tetraether lipids which are less susceptible to acid hydrolysis **(b)** Presence of putative proton efflux system that includes secondary transporters ATPases, antiporters and symporters **(c)** high number of putative cation transporters, possibly involved in the generation of Donnan potential **(d)** carry genes encoding the enzymes of organic acid degradation **(e)** contain cytoplasmic buffering molecules which have capacity to sequester protons **(f)** presence of heat shock proteins/chaperones and quick DNA repair system.

Despite being able to survive in extreme acidic conditions, intracellular pH of acidophiles is similar to that of neutrophiles because macromolecules such as DNA become unstable at acidic pH. Their pH gradients [pH gradient (pH) = pH_in_ − pH_out_], however, remain several orders of magnitude greater than neutrophiles. In order to survive, acidophiles have evolved multiple mechanisms such as highly impermeable cell membranes, small genomes for ease in maintenance, genes for organic acid degradation, DNA and protein repair systems, and a predominance of secondary transporters to remove protons once they have entered cytoplasm (Figure 2) (Johnson, 1998; Baker-Austin and Dopson, 2007; Sharma et al., 2016). Acidophiles are the most widely distributed in the bacterial and archaeal domains (Table 1) and have numerous biotechnological applications (Sharma et al., 2012; Elleuche et al., 2014; Mehta and Satyanarayana, 2016).

## α- amylase producing acidophiles

Despite extensive research on acidophiles, very few have been exploited for commercial purposes. Matzke et al. (1997) reported α-amylase from acidophilic bacterium *Alicyclobacillus acidocaldarius*, which is thermostable and acidstable with a molecular mass of 140 kDa, with optimum temperature and pH of 75°C and 3.0, respectively. Bai et al. (2012) reported another α-amylase from *Alicyclobacillus* sp. A4 with a molecular mass of 64 kDa and optimal activity at 75°C and pH 4.2. In still another report, an α-amylase from the acidophilic bacterium *Bacillus* sp. DR90, isolated from Dig Rostam hot mineral spring (Iran), was investigated. The enzyme was active in a wide range of pH and temperature having optimal activity at pH 4.0 and 75°C with a molecular mass of 76 kDa (Asoodeh et al., 2014). Laderman et al. (1993) reported a thermoacidophilic α-amylase from *Pyrococcus furiosus* which was optimally active at ~100°C and pH 5.5–6.0. The enzyme is a homodimer with a subunit molecular mass of 66 kDa. Recently a Ca^2+^-independent, acid-stable α-amylase (Ba-amy) from the acidophilic bacterium *Bacillus acidicola* TSAS1 has been investigated in detail. This has been found to be a potential candidate for saccharification of starch at its native pH of 3.0–4.5 with T_1/2_ of 25 min at 70°C (Sharma and Satyanarayana, 2010).

## Production of acid-stable amylases

At commercial level, amylolytic enzymes are being produced from *Bacillus* spp. (*B. licheniformis, B. stearothermophilus and B. amyloliquefaciens*) and filamentous fungi such as *Aspergillus oryzae* and *A. niger* (http://www.novozymes.com/) (Sharma and Satyanarayana, 2013a; Sharma et al., 2016). They are the preferred sources because of their rapid growth rates, economical production and ability to secrete a large quantity of amylases, which lack either acid stability or thermostability. Amylases from acidophiles have tremendous potential to replace neutrophilic enzymes because they are active in the acidic range. Production on a commercial scale is, however, a major bottleneck because of low titres of extracellular amylases secreted by acidophilic and acid tolerant microbes (Table 4). Schwermann et al. (1994) recorded maximum amylase production (90 U mL^−1^) in the presence of maltose as compared to other carbon sources used in the production medium. Bai et al. (2012) reported a 2.3 U mL^−1^ α-amylase production in *Alicyclobacillus* sp. A4 after 48 h in the presence of starch as carbon source. A few researchers have attempted multiple strategies to increase the extracellular acid-stable amylase titres (Table 4). Kanno (1986) improved *A. acidocaldarious* A2 strain by using UV/enrichment method for α-amylase production. A total of 11-fold increase in the enzyme titre was achieved as compared to the wild type strain (220 U mL^−1^). Brown et al. (1990) attained a low constitutive level of α-amylase from *Pyrococcus furiosus* in the presence of simple sugars, while polysaccharides with α-1,4 linkages stimulated production. The maximum yield of extracellular α-amylase was achieved (100 UmL^−1^) in *P. furiosus* in the presence of pullulan as a carbon source in submerged fermentation at 98°C.

A detailed investigation was carried out on the production of α-amylase by *B. acidicola* in submerged fermentation. Conventional “one-variable-at-a time” and statistical approaches have been used for optimizing the cultural parameters (Sharma and Satyanarayana, 2011). α-Amylase production by *B. acidicola* was high in the presence of soluble starch (2%) as a carbon source. Among nitrogen sources tested, tryptone (HIMEDIA) [0.5%] in combination with yeast extract (0.5%) supported a high enzyme titre. The α-amylase produced by *B*. *acidicola* displayed a high activity (8 U/mL) at pH 4.0 and 37°C after 44 h. The enzyme production was high when the cells are in stationary phase as in *Geobacillus thermoleovorans* (Uma Maheswar Rao and Satyanarayana, 2003). Further statistical approaches [Plackett-Burman design and Response Surface Methodology (RSM)] were employed for optimizing α-amylase production by *B. acidicola* in submerged fermentation. When the effect of 11 variables on α-amylase production was assessed using Plackett-Burman design, four variables (starch, K_2_HPO_4_, inoculum size, and temperature) were identified to significantly affect enzyme production. In order to control process parameters like aeration, uniform distribution of nutrients, and heat and oxygen transfer for α-amylase production by *B. acidicola*; fermentation was carried out in a 7 L laboratory fermentor. A reduction in fermentation time for attaining the peak was recorded; this could be due to improvement in mixing of nutrients and the control of dissolved oxygen (Kumar and Satyanarayana, 2007). Further, a 2.9-fold enhancement in enzyme production was attained due to fed-batch fermentation as compared to that in the initial unoptimized medium (3.5 U mL^−1^) (Sharma and Satyanarayana, 2011).

*Bacillus acidicola* is amenable to solid state cultivation like some other *Bacillus* spp. (Babu and Satyanarayana, 1995; Chen et al., 2005). Therefore, production of α-amylase by solid state fermentation was also attempted in order to find the prospects of using a wide range of agro-industrial residues as substrates (Babu and Satyanarayana, 1995; Sharma and Satyanarayana, 2012b). A peak in enzyme production was reached in 72 h, when 10 g of wheat bran was used in 250 mL Erlenmeyer flasks as reported for the production of α-amylase by *Bacillus coagulans* (Babu and Satyanarayana, 1995). In contrast, 5 g wheat bran per 250 mL flask was used for α-amylase production by *B. amyloliquefaciens* (Gangadharan et al., 2006). *Bacillus acidicola* secreted high enzyme titre at a_w_ (water activity) 0.95; below this, α-amylase production declined, with no growth and enzyme production below a_w_ 0.85, indicating that the bacterium is desiccation sensitive. Supplementation of wheat bran with ammonium sulfate supported a high enzyme titre. The addition of nitrogen sources to the solid substrates had been shown to enhance the production of various enzymes including α- amylase in solid state fermentation (SSF) (Pedersen and Nielsen, 2000). Moisture (substrate:water, 1:3.5), starch (2.9%) and ammonium chloride (0.38%) supported a high enzyme titre in *B. acidicola*. Statistical optimization of α-amylase production in SSF using response surface methodology led to 5.6-fold [28 ± 2.3 U/g dry bacterial bran (DBB)] increase in the titre as compared to unoptimized conditions (5 ± 1.1 U/g DBB) (Sharma and Satyanarayana, 2012b).

## Cloning and expression of acid-stable α-amylase encoding genes

Despite extensive efforts, attaining higher enzyme titres by wild type microbial strains is a major hurdle for their commercialization. By cloning and expressing acid-stable amylase encoding genes from acidophiles in mesophilic hosts such as *E. coli, Pichia pastoris* or *Bacillus subtilis*, the production can be made cost-effective (Table 4). However, improper folding and differences in codon usage often hinder over production of enzymes in heterologous expression systems (Sharma et al., 2012). In order to overcome these bottlenecks, researchers use codon optimized synthetic genes or different host variants (Elleuche et al., 2014; Ranjan and Satyanarayana, 2016). Acid-stable α-amylase gene from *Bacillus* sp. DR90 was successfully cloned in *E. coli* BL21 and expressed as an intracellular active protein. After induction, specific activity was around 600 U/mg (Asoodeh et al., 2010). Matzke et al. (1997) cloned acid-stable α-amylase gene from *Alicyclobacillus acidocaldarius* in *E. coli*. The enzyme was intracellular and the optimum temperature for recombinant acid-stable α-amylase was slightly lower than that of the native enzyme. Extracellular acid-stable α-amylase encoding gene of *P. furiosus* (PFA) was cloned and expressed in *E. coli* (Dong et al., 1997). The recombinant acid-stable α-amylase was mainly expressed in the form of insoluble inclusion bodies. An improved purification method was developed by Wang et al. (2007). The solubilization of the inclusion bodies was achieved by treatment at 90°C for 3 min in Britton–Robinson buffer at pH 10.5. After solubilization, a total of 58,000 U/g wet cells yield was obtained. In another study, Peng et al. (2016) co-expressed PFA with chaperones in *E. coli*. Both chaperonin and a small heat shock protein (sHSP) increased the solubility of PFA to a certain degree, while pre-folding seemed to be the most efficient that increased the enzyme activity to about 60,000 U g^−1^ wet weight over that of 5,000 U g^−1^ wet weight without chaperone. Wang et al. (2016) produced soluble PFA by expressing PFA in *B. amyloliquefaciens*. The yield of PFA was 2,000 U mL^−1^ of supernatant and 2,714 U mL^−1^ of total culture. Zhu et al. (2017) expressed PFA in *Nicotiana tabacum* and found that plant produced PFA forms functional aggregates with an accumulation level up to 3.4 g kg^−1^ fresh weight. The aggregates were functional without requiring refolding. As stated above, several attempts have been made to increase acid-stable α-amylase titres. Intracellular accumulation and inclusion body formation make them impractical for industrial applications (Grzybowska et al., 2004; Wang et al., 2007).

A truncated 1,441 bp acid-stable α-amylase gene encoding 479 amino acid α-amylase (Ba-amy) of *B. acidicola* was successfully cloned and expressed in active form in *E. coli*. Various approaches have been developed for efficient secretion of proteins such as increasing the permeability of the outer membrane chemically (adding EDTA, glycine, and Triton X-100) and by enzymatic (lysozyme) treatments (Sharma and Satyanarayana, 2012a; Parashar and Satyanarayana, 2016a). The purified recombinant α-amylase was active at pH 4.0 and 60°C, and retained all characteristics like that of the native α-amylase.

The methylotrophic yeast *Pichia pastoris* has emerged as an important production host for extracellular production of proteins for both basic research and industrial applications (Cregg et al., 2009; Spohner et al., 2015). Codon usage analysis of acid-stable *Ba-amy* revealed the feasibility of its expression in *Pichia pastoris*. In order to increase extracellular production of acid-stable α-amylase, *Ba-amy* was cloned and expressed in *P. pastoris* under dual promoters (*GAP* and *AOX*) and fused with α-factor secretion signal peptide. Mixed fed batch and high cell density cultivation experiments were performed which led to 15- and 7- fold higher extracellular enzyme titres than that of the wild type *B. acidicola* and recombinant *E. coli*, respectively (Parashar and Satyanarayana, 2016b). The recombinant acid-stable Ba-amy purified from *P. pastoris* was biochemically characterized, which revealed kinetic properties and thermostability of glycosylated acid-stable Ba-amy to be similar to those of the recombinant acid-stable Ba-amy expressed in *E. coli*. The engineered Ba-amy (Ba-Gt-amy) was also cloned and expressed in *P. pastoris* (Parashar and Satyanarayana, 2017a). The combination of multiple transformations and post-transformational vector amplification (PTVA) and high cell density cultivation in fermentor led to a very high production (750 U/mL) of the chimeric Ba-Gt-amy (Parashar and Satyanarayana, 2017a).

## Structural characteristics of acid-stable α-amylase

Although the adaptation of acidstable enzymes to low pH has not been explored in greater detail, one explanation for pH stability has been offered from the modeling of α-amylases from *B. acidicola* and other acid-stable amylases. A detailed investigation revealed that the acid stability and activity at acidic pH could be attributed to the surface charge density and amino acid composition of these proteins (Table 2). A prominent feature of acidstable α-amylases is the excess of glutamic and aspartic acid (D + E) residues on their surface as compared to their closest relatives. Moreover, the enzyme contains less positively charged amino acid residues (K + R + H) than their neutrophilic counterparts that leads to reduced positive charge density at the surface of the protein (Figure 2)(Reed et al., 2013). This effect was interpreted as follows: if the proteins were to possess a large content of positively charged residues (K + R), the positive charges at the surface will repel each other, leading to unfolding of the protein. On the other hand, protonation of the negatively charged group increases at lower pH that leads to reduction in the negative charge, which aids in stabilizing proteins in acidic conditions. If such proteins were to possess a large excess of negative groups, unfolding might also occur above the isoelectric point of the protein due to disruption of stabilizing structural interactions. In order to be stable and active in a broad pH range, these groups of proteins must have reduced number of D + E residues, which is compensated by an increase in the number of polar residues (Matzke et al., 1997; Schäfer et al., 2004; Reed et al., 2013). These characteristics were also found in other proteins. Huang et al. (2005) reported a high number of acidic residues on the surface of proteins, which causes repulsion due to excess negative charges resulting instability of proteins at high pH. However, a few exceptions have been found to this rule. Schäfer et al. (2004) reported thermo-acid-stable maltose-binding protein from *Alicyclobacillus acidocaldarius*. This protein has higher content of basic residues exposed on its surface, while most acidic residues are buried in the interior. As a consequence, this protein has a highly positive surface charge. This study suggested that there are multiple factors responsible for the acid-stability of proteins.

**Table 2:** Amino acid composition (%) and other characteristics of acidic, basic and neutral amylases.

**Characteristics**	**Basic amino acid (H + R + K)**	**Acidic amino acid (D + E)**	**Total amino acid**	**PI**	**Molecular weight**	**Source**	**Reference**
Acidic	6.4	10.5	1276	4.36	137	*Alicyclobacillus acidocaldarius*	Matzke et al., 1997
Acidic	11.1	10.9	626	5.53	68	*Bacillus* sp. *DR90*	Asoodeh et al., 2014
Acidic	11.7	11.9	479	5.41	62	*B. acidicola*	Sharma and Satyanarayana, 2010
Acidic	10.4	12.9	435	4.82	50	*Pyrococcus furiosus*	Laderman et al., 1993
Basic	10.5	15.2	923	4.61	103	*Bacillus halodurans*	Murakami et al., 2007
Basic	9.3	15.5	922	4.44	102	*Bacillus* sp.	Shirokizawa et al., 1990
Neutral	15.4	12.9	483	6.05	55	*Bacillus licheniformis*	Joyet et al., 1984
Neutral	15.7	15.7	488	5.62	57	*Geobacillus thermoleovorans*	Mehta and Satyanarayana, 2014

*H, Histidine; R, Arginine; K, lysine; D, aspartic acid; E, Glutamic acid; pI, isoelectronic point*.

Another theory, a change in the pH activity of the amylases is depends the pKa of the catalytically important residues which are known to be influenced by the electrostatic field. It has been hypothesized that slight change in the pKa values of the catalytic residues can change the pH activity profile of the enzyme (Nielsen et al., 1999; Nielsen and Borchert, 2000). The pKa of catalytic residues in the active site can be altered by mutating selected residues that can alter the hydrogen bonding network, solvent accessibility or change in the net charge of the molecule (Nielsen and Borchert, 2000). This can be explained as follows: active site residues must be in a catalytically competent protonation state for the enzyme to be active. Thus the proton donor (Glu) is required to be protonated, while the nucleophile (Asp) must be negatively charged. If an α-amylase is stable over the entire pH range, it is feasible that the pH-activity profile can be changed if the pKa value of either the nucleophile or the proton donor is changed. Typically, charged residues are inserted in the vicinity of the titrable group to change the immediate environment of the active site of enzymes (Wind et al., 1998; Nielsen et al., 1999; Nielsen and Borchert, 2000). In several cases, it has been observed that pH ± activity profiles shifted in the opposite direction as compared to the shift predicted from electrostatic calculations. This strongly suggests that electrostatic effects cannot be the best method to alter the optimum pH for enzymes. Secondary structural content does not appear to vary greatly in different amylases, which suggests that it is not a contributing factor.

**Figure 3:**
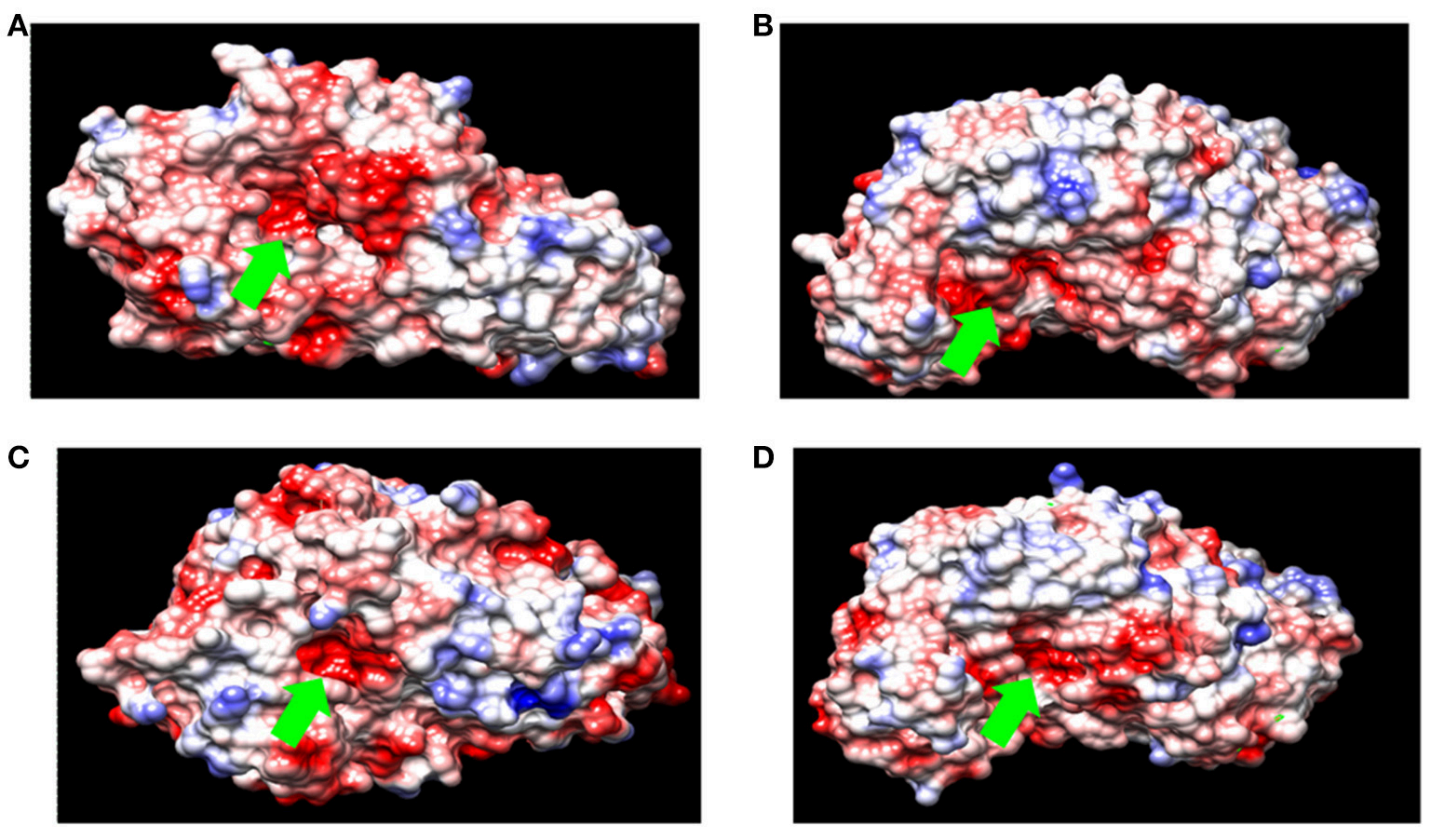
Surface charge density of **(A)** α-amylase from *B. acidicola* **(B)** α-amylase from *Bacillus* sp. DR90 **(C)** α-amylase from *B. licheniformis* **(D)** α-amylase from *Geobacillus thermoleovorans*. (red for negative potential, white near neutral and blue for positive potential. Green arrow indicates catalytic active site of amylase).

**Figure 4:**
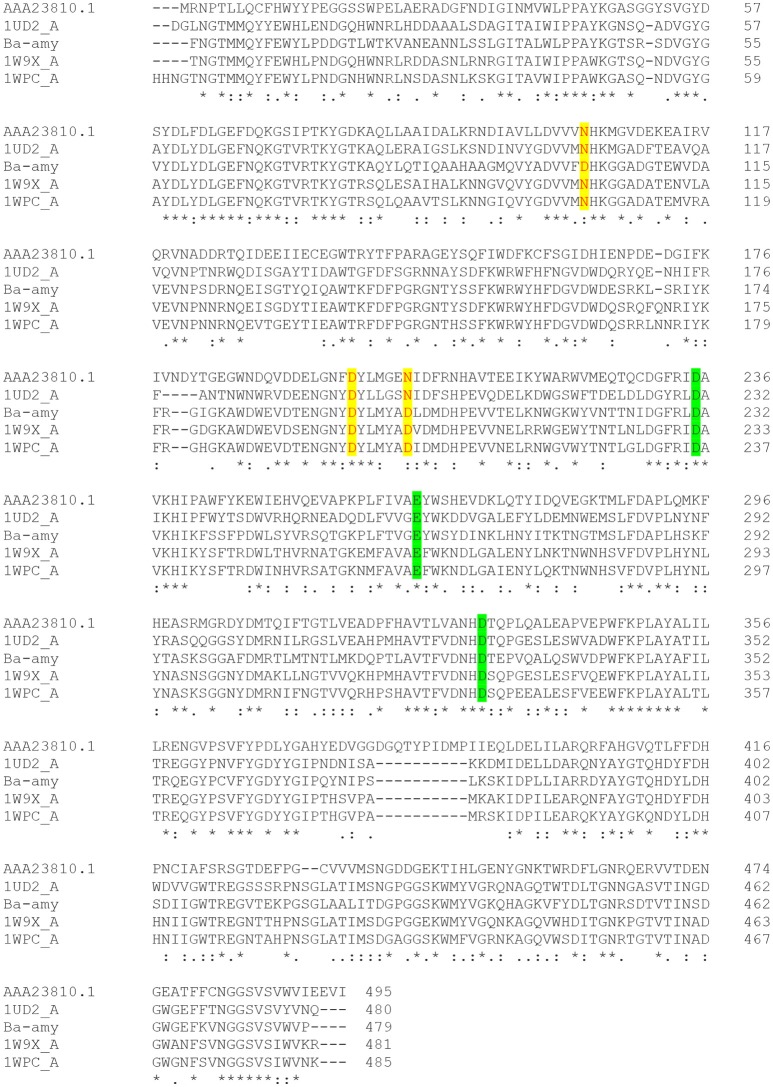
Multiple sequence alignment of different α-amylases. Residues responsible for calcium binding sites are highlighted in yellow color. Catalytically important residues are shown in green color. AAA23810.1- α-amylase from *Escherichia coli*; 1UD2_A: Calcium free α-amylase from *Bacillus* Sp. Strain Ksm-K38; Ba-amy: α-amylase from *B. acidicola*; 1W9X_A: *Bacillus halmapalus* α-amylase; 1WPC_A: maltohexaose producing α-amylase from *Bacillus* sp. 707. [(^*^) the residues are identical in all sequences; (:) the conserved substitutions; (.) semi-conserved substitutions].

Acid-stable α-amylases appear to degrade starch essentially by the same mechanism as neutrophilic α-amylases despite their distinguishing characteristics (Table 3). This contention is based on the deduction that the α-amylase from acid-stable and neutrophilic members conserved the same charge at the catalytic active site (Figure 3). Moreover, multiple amino acid alignments and site directed mutagenesis revealed that acid-stable amylases conserved same catalytic residues (Asp189, Glu320, and Asp401) like that of their neutrophilic counterparts (Figure 4). Hence, the acid stability of amylases has minor effect on their catalytic sites (Sharma and Satyanarayana, 2013b).

**Table 3:** Distinguishable properties of acid-stable and neutral α-amylases.

**Characteristics**	**Acidstable α-amylases**	**Neutral α-amylases**
pH range	3.0–6.0	6.5–8.0
Temperature range (°C)	40–115	37–90
Molecular weight (kDa)	41–160	12.5–70
pI	3.4–4.8	5.0–7.1
Acid-stablity	3.5–5.5	Unstable
Thermostability (°C)	60–80	Unstable
Release of CNP from CNP-α-G3	G3 Suppressed by KSCN	Stimulated by KSCN
Cleavage of G5	GGG(α) + GG	GG + GGG(α/β)
Number of subsites	5	7–9

*Modified from Sharma and Satyanarayana (2013a)*.

**Table 4:** Production profile of wild and recombinant acid-stable amylases from various acidophiles.

**Source**	**Optimum pH**	**Production by the wild strain U mL^−1^**	**Production in the recombinant *E. coli* (U mg^−1^)**	**Production in the recombinant *P. pastoris* U mL^−1^**	**Production in *Bacillus* spp. U mL^−1^**	**Production in plant**	**References**
*Alicyclobaillus* sp. *A4*	4.2	2.3	–	–	–	–	Bai et al., 2012
*Alicyclobacillus acidocaldarious*	–	–	0.33	–	–	–	Schwermann et al., 1994
*Alicyclobacillus acidocaldarius*	–	0.1	–	–	–	–	Schwermann et al., 1994
*B. acidocaldarius*	–	2200.0	–	–	–	–	Kanno, 1986
*Bacillus* sp. *DR90*	4	–	600.0	–	–	–	Asoodeh et al., 2014
*P. furiosus*	5.5	–	138.0	–	–	–	Wang et al., 2007
*P. furiosus*	5.5	100.0	–	–	–	–	Brown et al., 1990
*P. furiosus*	5.5	–	–	–	2714	–	Wang et al., 2016
*P. furiosus*	5.5	–	–	-	–	34.0 g Kg^−1^ fresh weight	Zhu et al., 2017
*P. woesei*	5.5	1.0	–	–	–	–	Koch et al., 1991
*B. acidicola*	4.0	12.0	180.0	750.0	–	–	Sharma and Satyanarayana, 2010, 2012a; Parashar and Satyanarayana, 2017a

## Protein engineering of α-amylases for acid stability and starch saccharification

Several methods of protein engineering are employed for improving acid stability of thermostable α-amylases to make them suitable for industrial applications, although success achieved so far is negligible. Nielsen et al. (1999) modified thermostable α-amylase from *Bacillus licheniformis* by using site directed mutagenesis, which was predicted to change the pKa values of the catalytic residues. The observations suggested that pH ± activity profiles of mutants which change the net charge on the molecule were significantly different from the wild-type pH ± activity profile. The differences were, however, difficult to correlate with the electrostatic field changes calculated. In another study, two amino acids of α-amylase from *B. licheniformis* were substituted (Leu134 to Arg and Ser320 to Ala) for acid tolerance, and the mutated gene was expressed in *Bacillus subtilis* WB600. The α-amylase variants were found to be more acid tolerant than the native protein. The optimum pH and stable pH range of the mutein (mutated protein) were 4.5 and 4.0–6.5, as compared to 6.5 and 5.5–7.0 as the optimum pH and pH stability range of the native protein. It has been postulated that mutations changed the net charge on the substituted residues, which influenced the pKa values of catalytically important amino acid residues (E261 and/or D328) (Liu et al., 2008a, 2012).

Yang et al. (2013) engineered amylase from *Bacillus subtilis* for improving protein stability and catalytic efficiency under acidic conditions by site-directed mutagenesis. Based on the analysis of three dimensional structure model, four basic histidine (His) residues (His222, His275, His293, and His310) in the catalytic domain were replaced with acidic aspartic acid (Asp) residues. The acid stability of the enzyme was significantly enhanced after mutation. It has been observed that the hydrogen bonds and salt bridges increased after mutation around the catalytic domain. The higher pKa of Asp was responsible for destabilizing the protonated form of Glu250, resulting in a decrease of the pKa value of Glu250. These changes around the catalytic domain have been suggested for improvement in protein stability and catalytic efficiency at low pH. Similar efforts have also been made for improving the pH stability of amylases by protein engineering (Shaw et al., 1999; Priyadharshini et al., 2010; Liu et al., 2012).

**Figure 5:**
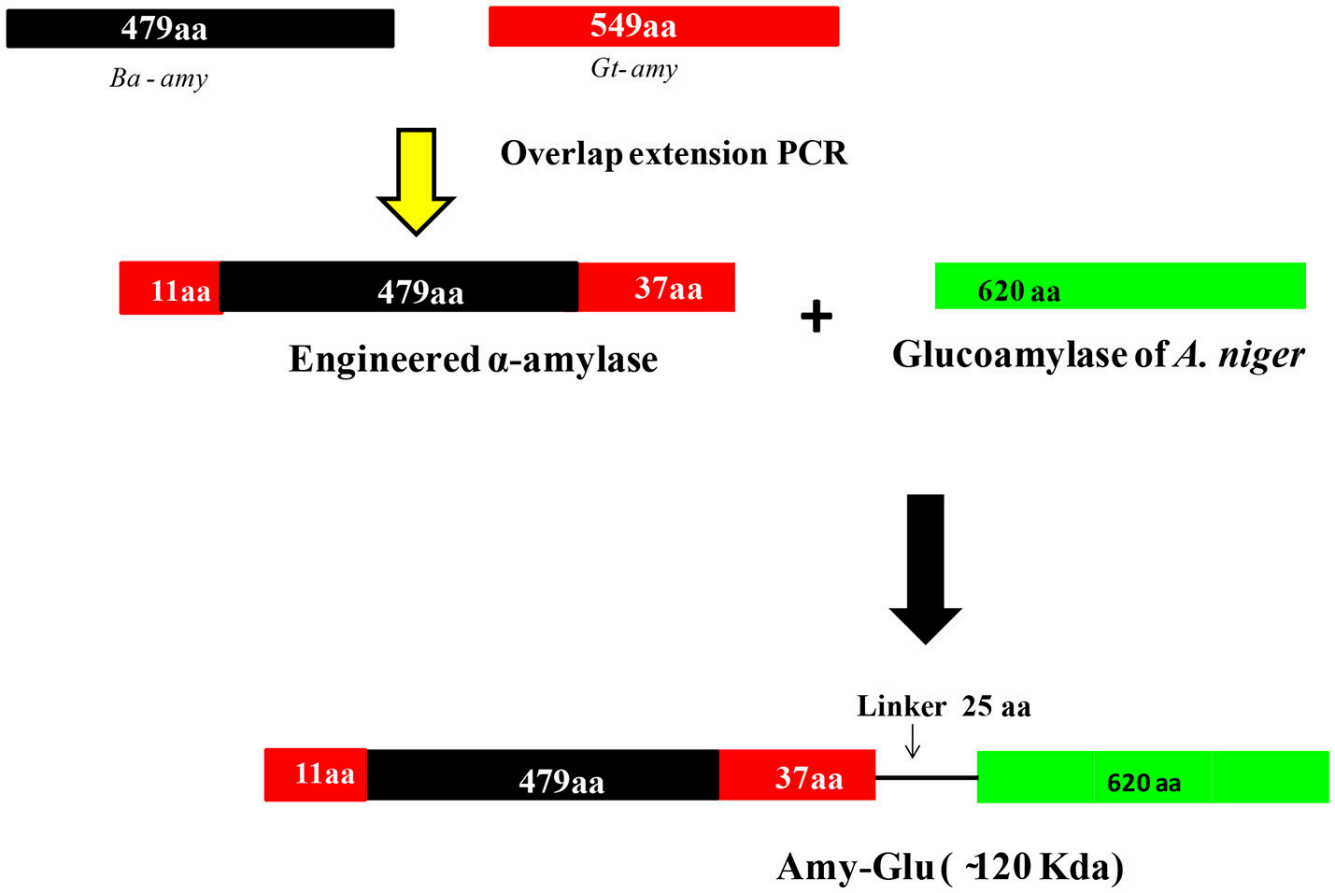
Strategy for generating different chimeras from α-amylase of *B. acidiola*. In the first step, amylase was engineered by adding 11 and 37 amino acids to N- and C- terminal ends from the α-amylase of *G. thermoleovorans*. In the second attempt, the engineered amylase and glucoamylase (from *Aspergillus niger*) were fused through a linker peptide of 25 amino acids [(Gly-Gly-Thr-Gly-Ser)_5_] {(Gly-Gly-Thr-Gly-Ser)_5_} (modified with permission from Parashar and Satyanarayana, 2016c, 2017a).

Despite a few strategies reported in the literature for improving acid stability of proteins (Liu et al., 2008a,b, 2012; Yang et al., 2013), there is no universal strategy that aids in engineering pH-activity profiles. Therefore, further research efforts are needed to find other factors that can contribute to the acid stability of proteins. Investigations have shown that it is possible to increase the thermostability, but not so with acid-stability. Therefore, instead of increasing acid-stability of already existing thermostable enzymes, a better option is to improve the thermostability of acid-stable enzymes. Since α-amylase from *B. acidicola* (Ba-amy) is stable in acidic conditions with moderate thermostability, an attempt has thus been made to improve thermostability (Parashar and Satyanarayana, 2016c). Several chimeras were constructed with the addition of amino acids at N- and C-terminal ends of acid-stable Ba-amy from the α-amylase of *Geobacillus thermoleovorans* (Gt-amy) (Figure 5). All chimeras were successfully expressed in active form in *E. coli*. Among all chimeras, one chimera displayed higher thermostability and specific activity as well as catalytic efficiency without change in its acid stability and pH optimum for activity (Parashar and Satyanarayana, 2016a). Increase in starch binding capacity of the chimeric α-amylase was observed in comparison with that of the wild type. The adsorption of chimeric α-amylase to raw starch suggests that the hydrolysis of raw starch can occur at high temperatures without energy-intensive gelatinization step, which brings down the energy consumption for starch saccharification. Furthermore, the end products of raw starch hydrolysis by the chimera suggested that the addition of residues did not alter the catalytic activity.

In another study, this chimeric α-amylase was fused with acid-stable glucoamylase of *Aspergillus niger* through a linker peptide for saccharifying starch in a single step (Figure 5) (Parashar and Satyanarayana, 2017b). The kinetic properties of the fused enzyme supported its suitability in raw starch saccharification in acidic conditions of native starch that liberates glucose besides maltodextrins as the major starch hydrolysis products. The fused chimeric enzyme can, therefore, be a practical option for the cost effective saccharification of raw starch. Engineering multidomain enzymes that are capable of catalyzing two or more reactions is a potential strategy to reduce enzyme costs in industrial processes because multiple catalytic properties in a single polypeptide simplify production and purification process (Fan et al., 2009; Ribeiro et al., 2011; Parashar and Satyanarayana, 2017b).

## Calcium binding region in α-amylase

Calcium is known to stimulate α-amylases and has also been implicated in enhancing their thermostability (Savchenko et al., 2002). Acid-stable Ba-amy was found to be a calcium-independent. A few Ca^2+^-independent α-amylases have also been reported earlier (Babu and Satyanarayana, 1993; Sajedi et al., 2005; Asoodeh et al., 2010), which were considered to be useful in industrial starch saccharification.

**Figure 6:**
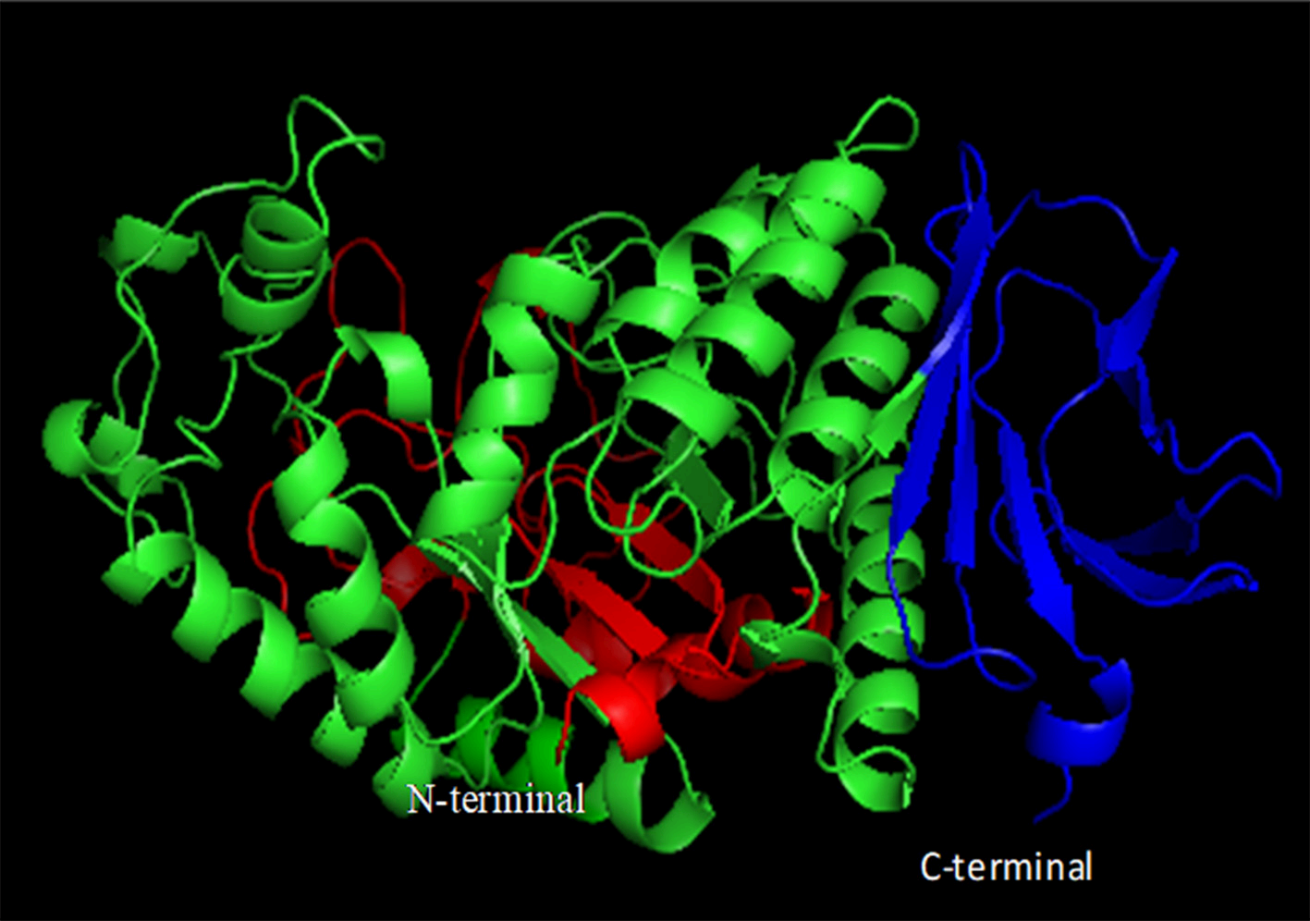
Domain organization of α-amylases. Domain A is shown in green, domain B in red, and domain C in blue (constructed using PyMOL).

In general, most of the α-amylases possess conserved calcium ion binding sites, which are positioned at the interface between domains A and B, and play a major role in its stability and activity (Figure 6) (Boel et al., 1990; Linden et al., 2003). *Bacillus* α-amylases have been reported to have three Ca^2+^ ions and one Na^+^ ion and a metal triad bridge (calcium-sodium-calcium) (Linden et al., 2003). This metal triad is important for maintaining the compact protein structure and provides thermal stability to the enzyme (Linden et al., 2003). Calcium ion helps in salting out of hydrophobic residues in the protein, resulting in the formation of a compact structure that enhances stability (Linden et al., 2003). Whenever calcium ions are detached, amylases lose their stability, while its restoration recovers the stability (Boel et al., 1990). Sequence analysis revealed that acid-stable Ba-amy comprises three calcium binding sites despite the fact that Ba-amy does not show any calcium-dependent activity. Since the calcium binding site is far away from the active site residues, it has been postulated that the role of calcium ions is in maintaining the structure rather than in catalysis. A second theory suggests that calcium is loosely bound, which is replaced by other metal ions such as Na^+^. These theories get support from the investigations on other calcium-independent enzymes (Nonaka et al., 2003).

## Commercial application of acidstable amylases

**Figure 7:**
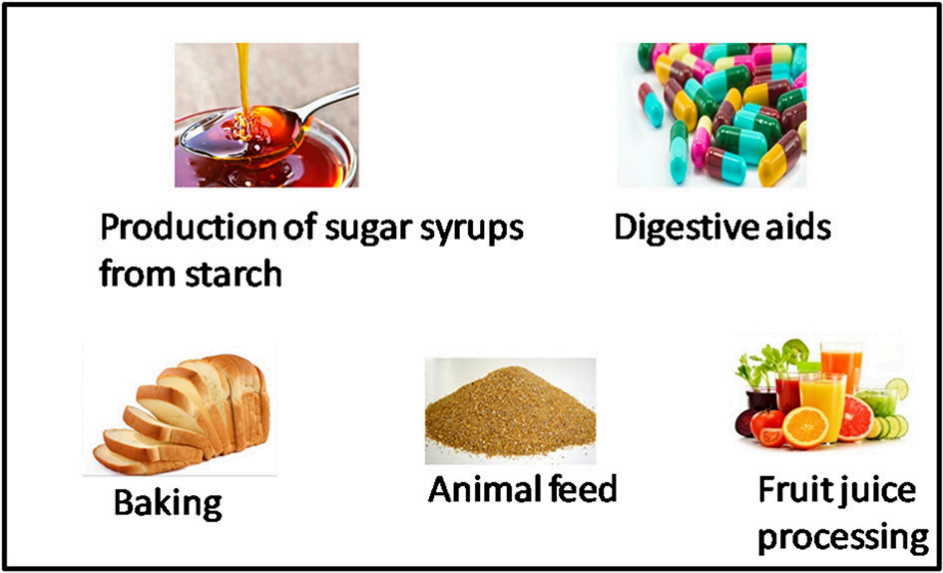
Applications of acid-stable α-amylases.

Extremophiles are potent sources of extremozymes, which display a high stability under extreme bioprocess conditions (Elleuche et al., 2014). Thus biocatalysts from extremophiles have been shown to be useful in industrial bioprocesses. Only a few extremozymes, however, found their way to the market (e.g., thermostable DNA polymerases from *Thermus aquaticus* and *Pyrococcus furiosus* and others). There is a tremendous potential for acidstable enzymes from acidophiles to revolutionize existing industrial processes and to make many novel applications possible (Figure 7) (Mehta and Satyanarayana, 2016; Sharma et al., 2016). Moreover, acidstable α-amylases reduce the cost and time required for multistep maltooligosachharide production from raw starches, which can be used as antistaling agents in baking industry (Parashar and Satyanarayana, 2016c). Other acid-stable enzymes have potential biotechnological and industrial applications (Figure 7).

## Conclusions

α-Amylases produced by acidophilic microorganisms find applications in industrial processes such as starch saccharification and hydrolysis of polysaccharides in plant biomass in bioethanol production. The analysis of structure of these proteins suggests that acid-stable biocatalysts differ in surface charge, amino acid composition, salt bridges, and hydrophobicity. There are very few successful attempts in improving acid-stability of enzymes through protein engineering. These studies lack adequate rational concept, thus, difficult to apply for other proteins. Extensive as well as intensive efforts are, therefore, called for attaining high titres of acid-stable α-amylases and to understand the mechanisms which make them functional at low pH for ameliorating the existing enzymes for novel industrial applications.

## Author contributions

All authors listed have made a substantial, direct and intellectual contribution to the work, and approved it for publication.

### Conflict of interest statement

The authors declare that the research was conducted in the absence of any commercial or financial relationships that could be construed as a potential conflict of interest.
